# Antioxidant Potential and Capacity of Microorganism-Sourced C_30_ Carotenoids—A Review

**DOI:** 10.3390/antiox11101963

**Published:** 2022-09-30

**Authors:** Inonge Noni Siziya, Chi Young Hwang, Myung-Ji Seo

**Affiliations:** 1 Division of Bioengineering, Incheon National University, Incheon 22012, Korea; 2 Research Center for Bio Material & Process Development, Incheon National University, Incheon 22012, Korea; 3 Department of Bioengineering and Nano-Bioengineering, Incheon National University, Incheon 22012, Korea

**Keywords:** C_30_ carotenoid, antioxidant, microorganism, production

## Abstract

Carotenoids are lipophilic tetraterpenoid pigments produced by plants, algae, arthropods, and certain bacteria and fungi. These biologically active compounds are used in the food, feed, and nutraceutical industries for their coloring and the physiological benefits imparted by their antioxidant properties. The current global carotenoid market is dominated by synthetic carotenoids; however, the rising consumer demand for natural products has led to increasing research and development in the mass production of carotenoids from alternative natural sources, including microbial synthesis and plant extraction, which holds a significant market share. To date, microbial research has focused on C_40_ carotenoids, but studies have shown that C_30_ carotenoids contain similar—and in some microbial strains, greater—antioxidant activity in both the physical and chemical quenching of reactive oxygen species. The discovery of carotenoid biosynthetic pathways in different microorganisms and advances in metabolic engineering are driving the discovery of novel C_30_ carotenoid compounds. This review highlights the C_30_ carotenoids from microbial sources, showcasing their antioxidant properties and the technologies emerging for their enhanced production. Industrial applications and tactics, as well as biotechnological strategies for their optimized synthesis, are also discussed.

## 1. Introduction

Carotenoids are a broad class of lipophilic pigments that are naturally synthesized by photosynthetic and nonphotosynthetic organisms, including plants and certain species of algae, bacteria, fungi, and yeasts [1]. De novo carotenoid synthesis has also been observed within certain species of arthropods such as aphids, cicadas, and stink bugs [2,3,4]. Although noncarotenogenic organisms such as humans and crustaceans cannot synthesize these compounds, they can obtain carotenoids from their diet and accumulate them in their tissues [1,5]. Carotenoids have antioxidant, anti-inflammatory, and anticancer properties attributed to their biochemical characteristics. These properties have led to a rise in the demand for carotenoids in the food, feed, cosmetic, and pharmacological industries, where they are employed as colorants, biofortification compounds, immunoregulators, and preventative supplements for conditions associated with metabolic syndrome [6,7].

Commercially available carotenoids are mainly obtained from plant extracts or chemical synthesis, both of which are not sustainable and can be detrimental to the environment. As such, there is an inclination toward safer, more environmentally friendly options for consumer products. Carotenoids from plant extracts are limited by seasonal and geographic conditions, whereas those obtained from chemical synthesis yield undesired byproducts and hazardous waste.

Conversely, microbial production is a practical alternative from a commercial and ecological perspective. Bacterial sources of carotenoids include nonphototrophic and phototrophic bacteria. Anoxygenic phototrophs are commonly found in aquatic environments and require solar energy for growth and metabolism, performing photosynthesis without evolving oxygen, whereas oxygenic phototrophs, namely Cyanobacteria, produce oxygen during the photosynthetic process [8]. Carotenoid production in microorganisms can occur to mitigate oxidative damage as part of the microbial preventative and responsive defense mechanisms against reactive oxygen species (ROS). Various bacterial strains contain distinct pathways for carotenoid production. The final products of these pathways have different structures depending on the pathways, enzymes, and environmental conditions.

Carotenoids generally possess a linear hydrocarbon backbone composed of C_5_ isoprenoid units present as six, eight, nine, and ten units, which are classified as C_30_, C_40_, C_45,_ and C_50_, respectively. C_40_ carotenoids are the most abundant in nature, and thus the most researched [9]. Carotenoids contain double bonds in their structures, which allow for numerous stereochemical arrangements, and account for their strong antioxidant potential [10]. The extent of the double bonds largely defines the carotenoid absorption of light and spectral properties, which typically fall between 400 and 550 nm, from violet to green light [11]. Within this range, carotenoid colors are detected from yellow to red to violet. However, unpigmented precursors are also present within carotenoid production pathways [12,13]. Within the carotenoid groups, C_30_ carotenoids are distributed among Euryarchaeota, Firmicutes, Cyanobacteria, Alphaproteobacteria, and Gammaproteobacteria [14]. Currently, few natural sources of C_30_ carotenoids are known, and they are more commonly found in Gram-positive bacteria such as *Lactiplantibacillus plantarum* [15], *Cytobacillus firmus* (originally named *Bacillus firmus*) [16], and *Staphylococcus aureus* [17].

The majority of carotenoid research is centered on the more naturally abundant C_40_ carotenoids, resulting in a gap in the knowledge of other carotenoid classes. Disseminating information on the production of C_30_ carotenoids with health benefits comparable to C_40_ carotenoids is advantageous in expanding the current market share of carotenoids. The antioxidant properties of C_30_ carotenoids, where similar or greater than their C_40_ counterparts, provide alternative sources of natural carotenoids with varying applications in the food, feed, and health sectors. This article discusses the current knowledge on microbial production of C_30_ carotenoids and their commercial applications considering their antioxidant content and potential in the food and pharmaceutical industries.

## 2. Microbial C_30_ Carotenoids and Their Derivatives

**Figure 1:**
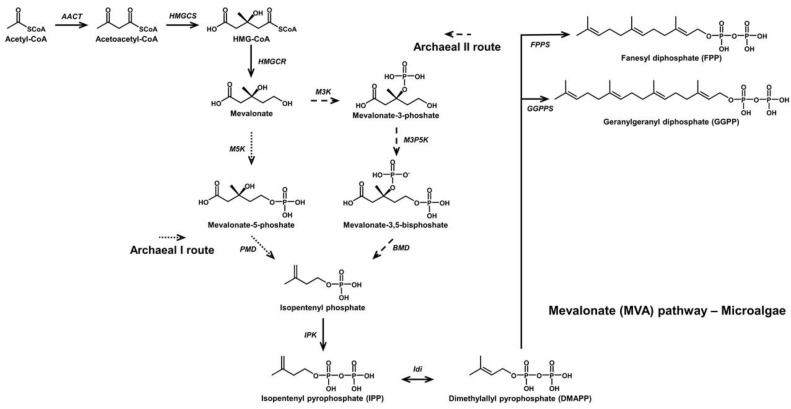
Mevalonate (MVA) pathway as taken by microalgae. AACT: Acetoacetyl-CoA thiolase. HMGCS: HMG-CoA synthase. HMGCR: HMG-CoA reductase. M3K: Mevalonate-3-kinase. M5K: Mevalonate-5-kinase. M3P5K: Mevalonate-3-phosphate-5-kinase. PMD: Phosphomevalonate decarboxylase. BMD: Bisphosphomevalonate decarboxylase. IPK: Isopentenyl phosphate kinase. Idi: Isopentenyl pyrophosphate isomerase. FPPS: Farnesyl pyrophosphate synthase. GGPPS: Geranylgeranyl pyrophosphate synthase.

**Figure 2:**
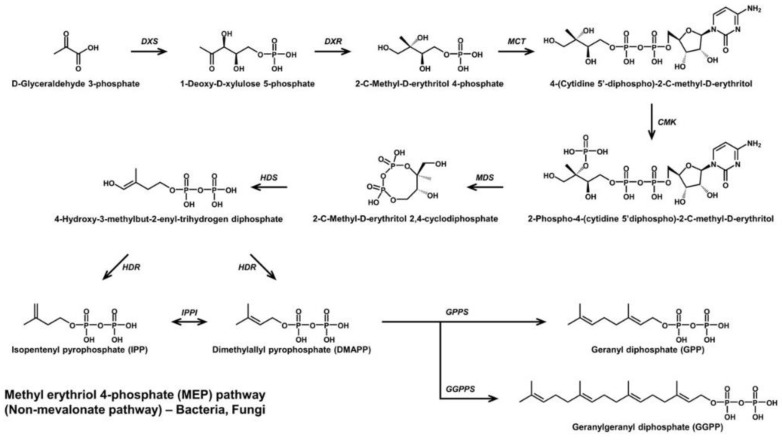
Methyl erythritol 4-phosphate (MEP) pathway as taken by bacteria and fungi. DXS: Deoxyxylulose 5-phosphate synthase. DXR: Deoxyxylulose 5-phosphate reductoisomerase. MCT: 2-C-methyl-d-erythritol 4-phosphate cytidylyltransferase. CMK: 4-diphosphocytidyl-2-C-methyl-D-erythritol kinase. MDS: 2-C-methyl-d-erythritol 2,4-cyclodiphosphate synthase. HDS: 4-hydroxy-3-methylbut-2-en-1-yl diphosphate synthase. HDR: (E)-4-hydroxy-3-methylbut-2-enyl diphosphate reductase. IPPI: Isopentenyl diphosphate isomerase. GPPS: Geranyl diphosphate synthase. GGPPS: Geranylgeranyl diphosphate synthase.

Like most carotenoids, the isoprene units that comprise C_30_ carotenoids are constructed from the isopentyl pyrophosphate (IPP) and dimethylallyl pyrophosphate (DMAPP) precursors from the mevalonate (MVA) pathway in plants and microalgae (Figure 1), and the methylerythritol phosphate (MEP) pathway in bacteria and fungi (Figure 2) [18]. The biosynthesis of C_30_ carotenoids occurs via either the condensation of two C_15_ farnesyl diphosphate (FPP) molecules, or the condensation of C_10_ geranyl diphosphate (GPP) and C_20_ geranylgeranyl diphosphate (GGPP). The former results in symmetric 4,4′-diapocarotenoids, whereas the latter produces asymmetric apo-8′-carotenoids [10,19,20].

**Figure 3:**
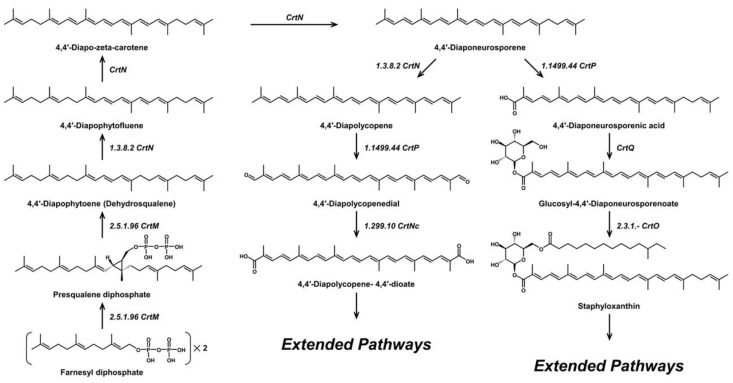
Biosynthetic pathway of 4,4′-diapocarotenoids. CrtM: 4,4′-Diapophytoene synthase. CrtN: 4,4′-Diapophytoene desaturase. CrtP: 4,4′-Diaponeurosporene oxidase. CrtNc: 4,4′-diapolycopene aldehyde oxidase. CrtQ: 4,4′-diaponeurosporenoic acid glycosyltransferase. CrtO: Glycosyl-4,4′-diaponeurosporenoate acyltransferase.

The C_30_ 4,4′-diapocarotenoids include 4,4′-diapocarotene-4-oic acid and fatty acid esters di-(β,D-glucosyl)-4,4′-diapocarotene-4,4′-dioate from *Methylobacterium rhodinum* (formerly *Pseudomonas rhodos*) [21]; 4,4′-diaponeurosporene and OH-diaponeurosporene from Heliobacteria [22,23]; and staphyloxanthin, 4,4′-diapophytoene, 4,4′-diapophytofluene, 4-4′-diapo-zeta-carotene, 4,4′-diaponeurosporene, and several of its derivatives from *S. aureus* [24,25] (Figure 3). Biosynthetic pathways of known carotenoids can be extended or altered through the inclusion of genes that further alter the forms of the carotenoids. Additionally, novel derivatives of these compounds can be produced by modifying reactions such as methylation, cyclization, and oxygenation.

**Figure 4:**
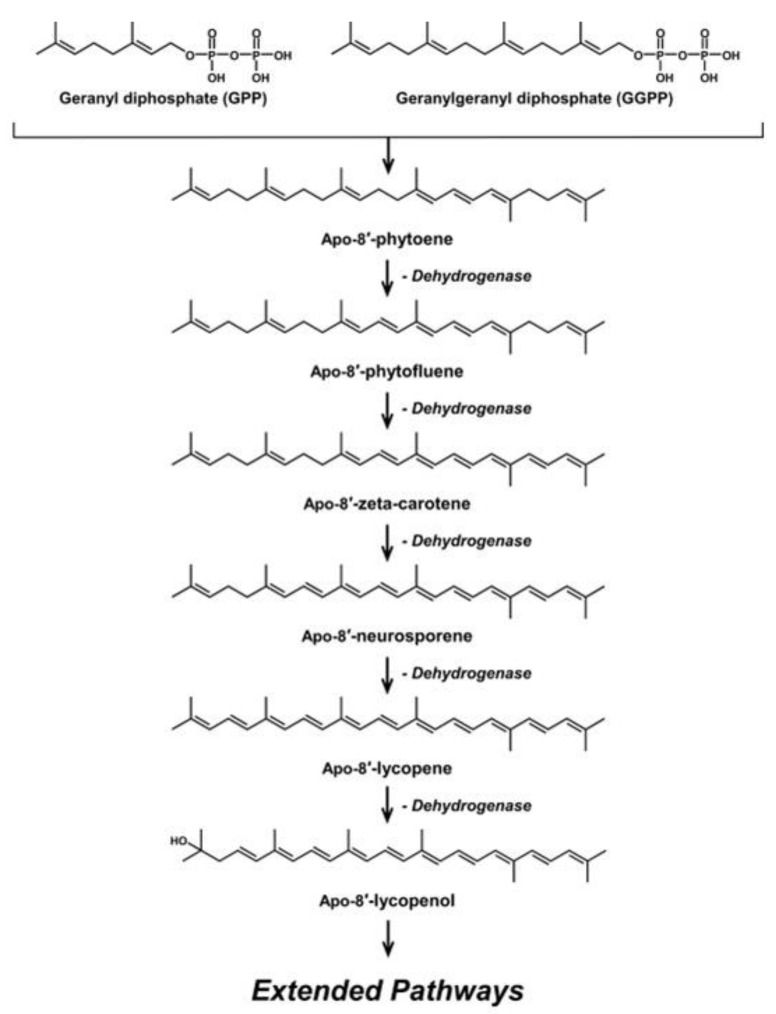
Biosynthetic pathway of Apo-8′-carotenoids.

The asymmetrical C_30_ apo-8′-carotenoids have three conjugated double bonds not found in the center of their skeletal structure (Figure 4), and include methyl glucosyl-3,4-dehydro-apo-8′-lycopenoate from *Planococcus* [26], and hydroxy-3,4-dehydro-apo-8′-lycopene and methyl hydroxy-3,4-dehydro-apo-8′-lycopenoate from *Halobacillus* [27]. Those of *Planococcus* were isolated products of natural biosynthesis beyond the known pathways. This suggests the possibility of gene clusters with unconfirmed genes that allow for their synthesis or other reactions within the bacteria. However, hydroxy-3,4-dehydro-apo-8′-lycopene and methyl hydroxy-3,4-dehydro-apo-8′-lycopenoate were produced after chemical mutagenesis of the microorganisms.

Different microbial C_30_ carotenoids originate from a limited number of microorganisms. Their properties are dependent on their structures, which can be altered further by changes or extensions in the biosynthetic pathways. Several C_30_ carotenoids are presented in Table 1 with their structures, bacterial sources, and antioxidant activities compared to those of C_40_ carotenoids and other standard compounds for the determination of antioxidant capacity.

4,4′-Diaponeurosporene (C_30_H_42_; also known as 7,8-dihydro-4,4′-diapo-ψ,ψ-carotene and *all-trans*-4,4′-diaponeurosporene) has been isolated from *S. aureus*, Heliobacteria, and *L. plantarum* subsp. *plantarum*, among others [17,22,28,29,30]. Research on the deep-yellow pigment revealed its potential against *Salmonella typhimurium* in a mouse model. The compound enhanced the immune system response and T-cell stimulation. It is also highly resistant to external stresses, consistent with its antioxidant potency [31]. These properties highlight its potential for medical or nutraceutical applications in immunocompromised individuals [30,32].

Colorless 4,4′-diapophytoene (C_30_H_48_; also called *all-trans*-4,4′-diapophytoene and dehydrosqualene) was the first C_30_ intermediate in the staphyloxanthin synthesis pathway [24,25]. Perez-Fons et al. [19] synthesized and isolated the C_30_ carotenoid apophytoene (C_30_H_48_) from spore-forming Bacillus species. Additionally, the C_30_ apocarotenoid derivatives of 1-glycosyl-3-4-dehydro-8′-apolycopene ester and methyl 1-glycosyl-3,4-dehydro-8′-apolycopenate ester were obtained in pigmented *Bacillus* species. The former was more abundant in vegetative cells, whereas the latter was dominant during spore formation. The carotenoids were considered apolycopene derivatives, which are different from those of diaponeurosporene (Table 1).

The bacteria *Planococcus maritimus* produces the acyclic C_30_ carotenoid methyl 5-glucosyl-5,6-dihydro-4,4′-diapolycopenoate, as well as C_30_ intermediates 5-hydroxy-5,6-dihydro-4,4′-diaponeurosporene, 5-glucosyl-5,6-dihydro-4,4′-diapolycopene, and 5-hydroxy-5,6-dihydro-4,4′-diapolycopene [33].

Staphyloxanthin (C_51_H_78_O_8_, also called 8′-apo-ψ,ψ-carotenoic acid) is a golden yellow pigment used to initiate immune system responses for the survival of infected host cells. The compound is more commonly associated with *S. aureus*, although several other species have been found to contain it, including *S. gallinarum* [34] and *S. carnosus*, from which it was identified as β-D-glucopyranosyl 1-O-(4,4′-diaponeurosporen-4-oate)-6-O-(12-methyltetradecanoate) [35].

**Table 1:** Antioxidant contents and capacities of C_30_ carotenoids.

C_30_ Carotenoid	Quenching Type	Antioxidant Activity		Conjugated Double Bond	Group Contained	H Bond Donor /Acceptor	Microbial Sources [Ref.]
		Carotenoid Content	Antioxidant Control				
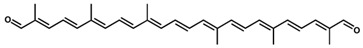 4,4′-Diapolycopenedial (4,4′-diapolycopen-4,4′-dial)/C_30_H_36_O_2_	DPPH scavenging	7.5 µM (IC_50_)	DL-α-tocopherol, 33.2 µM (IC_50_)	13	Aldehyde (2)	0/2	*Staphylococcus* spp. [36], *Methylomonas* spp. [37]
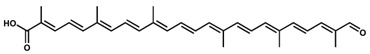 4,4′-Diapocaroten-4′-al-4-oic acid/C_30_H_36_O_3_	-	-	-	13	Carboxylic acid, aldehyde	1/3	*Pseudomonas* *rhodos* [21]
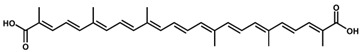 4,4′-Diapolycopene-4,4′-dioic acid/C_30_H_36_O_4_	Singlet oxygen quenching	5.8 µM	Astaxanthin, 9.3 µM	13	Carboxylic acid (2)	2/4	*Cytobacillus* *firmus* [16]
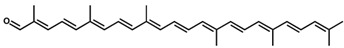 4,4′-Diapolycopen-4-al (4, 4′-Diapolycopenal)/C_30_H_38_O	-	-	-	12	Aldehyde	0/1	*Staphylococcus* *aureus* [24]
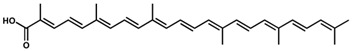 4,4′-Diapocarotenoic acid/C_30_H_38_O_2_	-	-	-	12	Carboxylic acid	1/2	*Pseudomonas* *rhodos* [21]
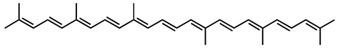 4,4′-Diapolycopene/C_30_H_40_	DPPH scavenging	8.7 µM (IC_50_)	DL-α-tocopherol, 33.2 µM (IC_50_)	11	Hydrocarbon	0/0	*Heliobacteria* spp. [36]
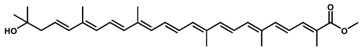 Methyl hydroxy-3,4-dehydro-apo-8′-lycopenoate /C_31_H_42_O_3_	Singlet oxygen quenching	44 μM (IC_50_)	Astaxanthin, 8.9 µM (IC_50_); β-carotene, >100 µM (IC_50_)	11	Ester, hydroxyl	1/3	*Halobacillus* *halophilus* [27]
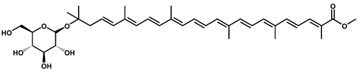 Methyl glucosyl-3,4-dehydro-apo-8′-lycopenoate /C_37_H_52_O_8_	Singlet oxygen quenching	5.1 μM (IC_50_)		11	Ester, glucosyl	4/8	*Planococcus* *maritimus* [26], *Halobacillus* *halophilus* [27]
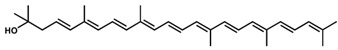 Hydroxy-3,4-dehydro-apo-8′-lycopene /C_30_H_42_O	Singlet oxygen quenching	79 μM (IC_50_)		10	Hydroxyl	1/1	*Halobacillus* *Halophilus* [27]
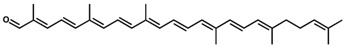 4,4′-Diaponeurosporen-4-al (Diaponeurosporenal)/C_30_H_40_O	DPPH scavenging	10.2 µM (IC_50_)	DL-α-tocopherol, 33.2 µM (IC_50_)	10	Aldehyde	0/1	*Staphylococcus aureus* [36]
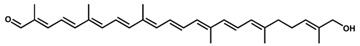 Hydroxy-diaponeurosporenal/C_30_H_40_O_2_	-	-	-	10	Aldehyde, hydroxyl	1/2	*Staphylococcus* *aureus* [38]
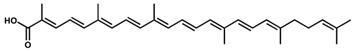 4,4′-Diaponeurosporen-4-oic acid/C_30_H_40_O_2_	DPPH scavenging	9.7 µM (IC_50_)	DL-α-tocopherol, 33.2 µM (IC_50_)	10	Carboxylic acid	1/2	*Staphylococcus* *aureus* [36]
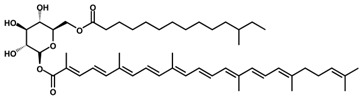 Staphyloxanthin/C_51_H_78_O_8_	DPPH scavenging	54.22 µg/mL (IC_50_)	Ascorbic acid, 35.54 µg/mL (IC_50_)	10	Ketone	3/8	*Staphylococcus* spp. [34]
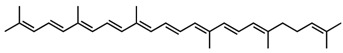 4,4′-Diaponeurosporene/C_30_H_42_	DPPH scavenging, singlet oxygen quenching	27.1% 11.6 µM (IC_50_) 45 µM	BHT, 50 µg/mL DL-α-tocopherol, 33.2 µM (IC_50_) astaxanthin, 3.7 µM	9	Hydrocarbon	0/0	*Staphylococcus* *aureus* [17], *Heliobacteria* spp. [22], *Lactiplantibacillus plantarum* [29,36]
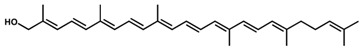 4-Hydroxy-4,4′-diaponeurosporene/C_30_H_42_O	-	-	-	9	Hydroxyl	1/1	*Streptococcus* *faecium* [39]
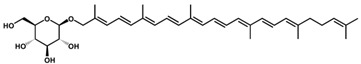 4-(D-Glucopyranosyloxy)-4,4′-diaponeurosporene/C_36_H_52_O_6_	-	-	-	9	Ester, glycosyl	4/6	*Streptococcus* *faecium* [39]
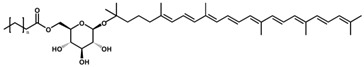 OH-Diaponeurosporene glucoside ester /C_40_H_60_O_7_	-	-	-	9	Ester	3/7	*Heliobacteria* spp. [23]
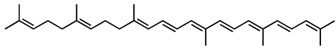 4,4′-Diapo-7,8,11,12-tetrahydrolycopene /C_30_H_44_	-	-	-	7	Hydrocarbon	0/0	*Staphylococcus* *aureus* [24]
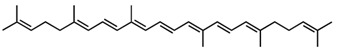 4,4′-Diapo-ζ-carotene (4,4′-Diapo-zeta-carotene) /C_30_H_44_	-	-	-	7	Hydrocarbon	0/0	*Staphylococcus* *aureus* [24]
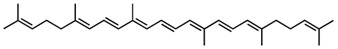 4,4′-Diapophytofluene/C_30_H_46_	-	-	-	5	Hydrocarbon	0/0	*Staphylococcus* *aureus* [24]
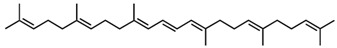 4,4′-Diapophytoene (Dehydrosqualene) /C_30_H_48_	-	-	-	3	Hydrocarbon	0/0	*Staphylococcus* *aureus* [24], *Bacillus* spp. [19]

Dashes (-) are designated as not yet studied or determined.

The orange pigment 4,4′-diapolycopene (C_30_H_40_; also known as 4,4′-diapo-ψ,ψ-carotene, *all-trans*-4,4′-diapolycopene) is an acyclic carotenoid produced in trace amounts by several species of anoxygenic photosynthetic Heliobacteria and by recombinant *B. subtilis* and *E. coli* [22,36,40].

4,4′-Diapolycopen-4-al (C_30_H_38_O; also known as *all-trans*-4,4′-diapolycopen-4-al, 4,4′-Diapo-ψ,ψ-caroten-4-al (International Union of Pure and Applied Chemistry, IUPAC), 4,4′-diapolycopenal and 4,4′-diapocaroten-4-al) is an apo carotenoid whose parent molecule is 4,4′-diapolycopene. 4,4′-Diapolycopen-4-al is found in trace amounts in *S. aureus* mutants [20,24,38,41]. 4,4′-Diapolycopenedial (C_30_H_36_O_2_; also known as 4,4′-diapo-ψ,ψ-carotenedial, 4,4′-diapolycopen-4,4′-dial, 4,4′-diapolycopene dialdehyde and *all-trans*-4,4′-diapolycopene-4,4′-dial) is a dialdehyde apocarotenoid also formed from 4,4′-diapolycopene. It has been isolated from *Staphylococcus* and methylotrophic bacteria [37,38].

Although a few studies have confirmed the isolation of C_30_ carotenoids, the antioxidant potential and microbial yields of these carotenoids can be underestimated owing to the difficulties faced during analysis. The identification of specific microbial-sourced carotenoids is hindered by their interactions with the materials used in standard protocols or by the limitations of separating intermediates and novel compounds that have variable characteristics from the more common carotenoid standards [42]. Low recovery and high carotenoid losses tend to occur due to their instability in the presence of light, heat, acids, alkali, and oxygen, as well as during chromatography. Therefore, all carotenoids necessitate special care in their handling. Additionally, stereochemical modifications influence their purification and analysis, as the *cis*/*trans* configurations affect their biochemistry and stereoisomerism affects their solubility and biofunctionality. *All-trans* isomers are predominant in naturally occurring carotenoids but are easily isomerized to their *cis* forms in the presence of light and heat. *Trans* forms have a propensity for crystallization due to their rigidity, and when converted to their *cis* isomeric forms, there is a slight reduction in the color saturation of the pigments [43,44,45].

Some carotenoids exhibit inconsistent retention in silica and octadecylsilyl (ODS) chromatography. To counter this problem, Osawa et al. [46] purified microbial carotenoid extracts using polystyrenic synthetic adsorbents and a CHP20/C10 packed column to separate the pigments. Their method was validated since the hydrophobic interactions between 4,4′-diapolycopene-4,4′-dioic acid and the polystyrene resin allowed for identification of the carotenoid using high-resolution electrospray ionization mass spectrometry (HRESI-MS) and nuclear magnetic resonance (NMR) analyses.

## 3. Antioxidant Properties of C_30_ Carotenoids

The antioxidant activity of a compound is the constant reaction rate between the compound and reactive species that include both radicals and nonradicals [13]. The physiological health benefits of carotenoids are attributed to the length of their conjugated backbones, which makes them highly reactive molecules capable of scavenging free radicals and quenching singlet oxygen. Carotenoids respond to ROS by donating electrons and, through oxidation, creating carotenoid–radical cations, thus lowering the oxidation state of the oxidizer [47]. Carotenoids also scavenge reactive oxygen species through the hydrogen atom transfer (HAT) mechanism in which a chemical transformation occurs, whereby there is movement of a proton and an electron between two substrates [48].

Currently, there are no universal measures for the determination of antioxidant activity. The antioxidant activity of C_30_ carotenoids is more commonly determined using the 1,1-diphenyl-2-picrylhydrazyl (DPPH) free-radical scavenging activity assay and the singlet oxygen quenching assay, which is performed by measuring the methylene blue-sensitized photooxidation of linoleic acid. While the DPPH assay is commonly used for antioxidant capacity determination with absorbance typically read at 515 nm, some carotenoids such as β-carotene and lycopene produce dark brown colors when mixed with DPPH. The color has been observed to interfere with the absorbance readings at 515 nm, but a change to 540 nm allowed for readings as there was still significant absorption at that wavelength [49,50]. The carotenoid antioxidant activity and reactivity in quenching ROS stem from the high electron density of the compounds, especially C=C bonds, and occur via physical or chemical ROS quenching. Singlet oxygen (^1^O_2_) originates from triplet molecular oxygen (^3^O_2_) in the presence of light, or superoxide and hydrogen peroxide in the absence of light. During physical quenching, carotenoids convert singlet oxygen to its triplet ground state, which is less reactive. The energy taken on by the carotenoid leaves it in an excited state, which dissipates as it releases the energy as heat into the environment and returns to its ground state. During chemical quenching (or scavenging), there is a chemical reaction between the carotenoid and the ROS, and barring enzymatic recycling of the carotenoid, it is oxidized and consumed completely. Carotenoid antiradical capabilities have also been studied by analyzing the carotenoid–hydrogen bond dissociation energy via the HAT antiradical mechanism [51]. Nakanishi et al. [52] developed a method to distinguish between hydrogen and electron transfer by using a galvinoxyl radical (G^●^) and magnesium ions. If electron transfer is the rate-determining step, the mechanism is accelerated by the magnesium via stabilization of the reduced radical anion. A subsequent proton transfer occurs from the radical cation, resulting in a neutral antioxidant radical and GH. However, information on this as applied to C_30_ carotenoids is lacking.

The structure of a carotenoid has significant bearing on its antioxidative efficiency. Depending on their structure, carotenoids can be classified as carotenes, which only have hydrocarbon backbones; and xanthophylls, which possess functional groups such as hydroxy, keto, and methoxy groups [53]. In certain microorganisms, the arrangement and yield of carotenoids are affected by their growth conditions, with different parameters eliciting different effects [54].

Stereoisomerism also plays a role in carotenoid biofunctionality and affects both solubility and absorbance. The rigidity of the *trans* forms increases their propensity for aggregation and crystallization. Additionally, certain *trans* forms have greater antioxidant activity than their *cis* counterparts, although exceptions such as *cis*-lycopene have greater bioavailability than *trans*-lycopene [55].

Carotenoids with higher numbers of double bonds tend to have higher scavenging activities, and acyclic compounds have greater scavenging activities than cyclic carotenoids [36]. Additionally, carotenoid functional groups influence antioxidant activity and solubility. Aldehyde groups increase carotenoid solubility, which in turn affects bioavailability and stability, two critical factors in carotenoid end-product manufacture.

Functional groups have a clear effect on C_30_ carotenoid reactivity. Scavenging activity is greater in carotenoids with aldehyde or carboxylic groups. Carboxyl groups have slightly higher antioxidant capacities than aldehyde groups, but aldehyde–protein crosslinking increases the timely effects of cosmetic applications, which improves the viability of commercial production [37]. Carbonyl and hydroxyl groups have significant roles in the potency of the quenching capacity of a carotenoid, which is attributed to the affinity of the carotenoid to the ROS and the hydrophobicity of the experimental solvent. As such, the antioxidant activities of carotenoids can be enhanced by increasing the number of conjugated double bonds (C=C and C=O) or by lowering the hydrophobicity of the compound through the addition of more hydroxyl groups [56].

According to Kim et al. [36], the activity of 4,4′-diapolycopene-4,4′-dial, which has 13 double bonds and two aldehyde groups, is greater than that of 4,4′-diaponeurosporene which only has 11 double bonds. Compared to DL-α-tocopherol, which had a DPPH half-maximum inhibitory concentration (IC_50_) value of 33.2 μM, the tested C_30_ carotenoids and their corresponding DPPH IC_50_ values were: diapolycopen-dial, 7.5 μM; diapolycopene, 8.7 μM; diaponeurosporenoic acid, 9.7 μM; diaponeurosporen-al, 10.2 μM; diaponeurosporene, 11.6 μM; diapotorulene, 70.3 μM; and diapo-β-carotene, 77.8 μM. Their double bonds were 13, 11, 10, 10, 9, 8, and 5, respectively. The cyclic compounds diapotorulene and diapo-β-carotene had one and two additional double bonds, respectively, within their β-end groups [36].

In another study, 4,4′-diaponeurosporene was isolated from *Lactiplantibacillus plantarum* subsp. *plantarum* KCCP11226^T^, a strain that showed high tolerance to oxidative stress, a high survival rate after H_2_O_2_ treatment, and a DPPH free-radical scavenging ability of 27.41%, which was greater than that of butylated hydroxytoluene (BHT, 50 μg/mL) [29]. Staphyloxanthin from *S. gallinarum* KX912244 has a DPPH free-radical scavenging activity of IC_50_ 54.22 µg/mL, a vitamin C equivalent antioxidant capacity (VEAC) while ascorbic acid had an IC_50_ value of 35.54 µg/mL [34].

Strong ^1^O_2_-quenching activity was observed in methyl 5-glucosyl-5,6-dihydro-4,4′-diapolycopenoate (5.1 µM), while the following intermediate carotenoids exhibited moderate ^1^O_2_-quenching activity compared to astaxanthin (3.7 µM): 5-glucosyl-5,6-dihydro-4,4′-diapolycopene (30 µM), 5-hydroxy-5,6-dihydro-4,4′-diapolycopene (30 µM), 4,4′-diaponeurosporene (45 µM), 5-hydroxy-5,6-dihydro-4,4′-diaponeurosporene (56 µM), and 15-*cis*-4,4′-diapophytoene (>100 µM) [33].

4,4′-Diapolycopene-4,4′-dioic acid and its methyl esters (monomethyl ester and dimethyl ester) were tested for their antioxidant activity according to their quenching capabilities of singlet oxygen (^1^O_2_). Their IC_50_ values were 5.8 μM, 6.0 μM, and 6.2 μM, respectively, compared to that of astaxanthin (9.3 μM) [46].

In addition to natural C_30_ carotenoids, several studies have reported the synthesis of novel C_30_ carotenoids, such as glycol-C_30_ carotenoids, which have different antioxidative properties. Mijts et al. [38] synthesized aldehyde and carboxylic acid C_30_ carotenoid derivatives along with 4,4′-diapolycopen-4,4′-dial using a carotenoid oxygenase (CrtOx, diapocarotenal synthase) from *S. aureus* to produce oxygenated linear C_30_ carotenoids. Methyl glucosyl-3,4-dehydro-apo-8′-lycopenoate (C_37_H_52_O_8_), a C_30_ carotenoid from *Halobacillus halophilus*, underwent chemical mutagenesis to isolate its intermediate compounds through a novel biosynthetic pathway. The intermediates, hydroxy-3,4-dehydro-apo-8′-lycopene and methyl hydroxy-3,4-dehydro-apo-8′-lycopenoate, were identified as 8′-apo derivatives that differed from 4,4′-diapo derivatives due to the nonsymmetric placement of the methyl groups rather than through FPP synthesis. Diapocarotenoid end groups are usually susceptible to oxidation by free peroxyl radicals formed in lipid membranes, possibly enhancing their reactivity [57,58]. The antioxidant activity of the three carotenoids was measured according to singlet oxygen quenching, with an IC_50_ value of 5.1 μM for methyl glucosyl-3,4-dehydro-apo-8′-lycopenoate, and 44 μM and 79 μM for its intermediates methyl hydroxy-3,4-dehydro-apo-8′-lycopenoate and hydroxy-3,4-dehydro-apo-8′-lycopene, respectively. The disparity between these three compounds was attributed to the lower hydrophobicity of the intermediate and to a greater number of conjugated double bonds ascribed to greater singlet oxygen quenching [27].

Shindo et al. [26] isolated methyl glucosyl-3,4-dehydro-apo-8′-lycopenoate, a C_30_ glycocarotenoic acid ester, from the marine bacterium *P. maritimus*. The singlet oxygen-suppressing activity of this carotenoid yielded an IC_50_ of 5.1 μM which was more potent than that of astaxanthin (8.9 μM) and β-carotene (>100 μM). Another marine bacterium, *Rubritalea squalenifaciens*, produces novel acyl glycocarotenoic acid called diapolycopenedioic acid xylosyl ester, which exhibits similar singlet oxygen suppression with an IC_50_ value of 5.1 μM. The esters isolated from this study were the first C_30_ carotenoids shown to have D-xylose groups, although others have reported acyl glucose within the compound structures. The potency of the antioxidant activity was attributed to the presence of carboxylic acids at either end of the aglycone structure [59].

The synthesis and isolation of rare and novel carotenoids from different types of bacteria, including lactic acid and marine bacteria, are steadily increasing the number of characterized carotenoids. Two novel C_30_ carotenoids, 4-[2-O-11Z-octadecenoyl-β-glucopyranosyl]-4,4′-diapolycopene-4,4′-dioic acid (IC_50_ 3.6 μM) and 4-[2-O-9Z-hexadecenoyl-β-glucopyranosyl]-4,4′-diapolycopene-4,4′-dioic acid (IC_50_ 3.2 μM), isolated from Methylobacterium strains, had antioxidative activities comparable to that of astaxanthin (IC_50_ 4.1 μM) and greater than that of β-carotene (IC_50_ >100 μM) [60]. Additionally, the antioxidant activity of the novel compound methyl 5-glucosyl-5,6-dihydro-apo-4,4′-lycopenoate yielded an IC_50_ of 5.1 μM from ^1^O_2_ suppression activity, while the IC_50_ values of astaxanthin and β-carotene were 8.9 μM and >100 μM, respectively [61].

## 4. Microbial Fermentation for Commercial Production of C_30_ Carotenoids

Only a few of the known carotenoids are commercially available, with most being C_40_ carotenoids such as astaxanthin and lycopene [11]. Chemical synthesis is the most common method used for industrial carotenoid production; however, it is associated with risks such as the generation of hazardous waste, and possible adverse effects include allergic reactions in humans [62]. Carotenoids obtained from plant extractions have environmental and geographic limitations and are relatively costly to purchase [63]. Microbial synthesis of carotenoids is increasingly preferable because of their sustainable nature, viable options for low substrate costs, simplicity of production, and monitoring of practices and applications [10,64].

A recent report on the global market for carotenoids stated that it was expected to reach USD 2.7 billion in 2027, increasing from USD 2.0 billion in 2022. This is consistent with their previous report on predicted growth. However, the majority of carotenoids are still supplied through chemical synthesis and are predominantly composed of C_40_ carotenoids such as β-carotene (C_40_H_56_), lutein (C_40_H_56_O_2_), astaxanthin(C_40_H_52_O_4_), and lycopene (C_40_H_56_). The C_30_ apocarotenoid, β-apo-8-carotenal (C_30_H_40_O), does have a part in the market value of carotenoids and can be sourced from the cyanobacterium *Arthrospira platensis* (or Spirulina), a microalgae species that can also synthesize various C_40_ carotenoids [65].

Carotenoid prices are dependent on origin and purity. Astaxanthin prices range from 2500 to 7000 USD/kg from microalgae *Haematococcus pluvialis*, but synthetic astaxanthin prices are typically less than 2000 USD/kg, with a production cost of only 1000 USD/kg, making them much more competitive and leaving the natural supply for more conscientous consumers who desire natural products [66,67]. β-carotene is priced between 300 and 3000 USD/kg when sourced from microalgae *Dunaliella salina* and has biomass production costs of about 17 USD/kg dry weight calculated from a process model [68,69,70]. Microalgae carotenoid production is highly costly, hindered by high capital, labor, and processing costs. Astaxanthin from *Haematococcus* has an estimated production cost of 718 USD/kg based on a conceptually designed facility, compared to costs of over 3000 USD/kg incurred by established firms producing the carotenoid from the microalgae [71]. Adaptive and recombinant microbial strains can reduce production costs in theory, but there is a lack of detailed economic assessment studies on carotenoid production by source that cover production, capital, labor, and processing costs. In addition, the studies that do tackle financial feasibility and requirements also focus on C_40_ carotenoids rather than other types.

Employing microorganisms as natural sources of carotenoids is beneficial to meet the growing demand for natural products. The short life cycles of microorganisms and lower land requirements for their production make them advantageous over plants for carotenoid extraction. In addition, many microorganisms can grow in stressful environments and use agricultural waste as nutrient sources, showcasing their environmental benefits. Microbes can be exploited to produce multiple desired products. The ease of genetic manipulation for enhanced microbial biosynthesis further highlights the potential of microbes for carotenoid production over chemical synthesis and plant extraction.

Carotenoids are lipid-soluble compounds typically extracted using organic solvents, with nonpolar solvents utilized for nonpolar compounds and polar solvents for polar carotenoids. Bacterial carotenoids can also be extracted by relatively costly enzymatic action. This process includes cell membrane hydrolysis in a greener, more sustainable process, although yield remains a concern. Another extraction method is microwave-assisted extraction, which uses microwave radiation to destroy cellular structures and release the desired product. Supercritical fluid extraction (SFE) is an improvement of traditional extraction as it uses water and carbon dioxide, thereby being more environmentally friendly. It also exhibits higher permeability and diffusivity, faster processing, and higher carotenoid extraction yields [44].

Utilizing microorganisms as commercial carotenoid sources is relatively cost-effective. However, the cost of substrates and running costs for large-scale production vary by country and region. Production costs are relatively lower in countries where substrate-manufacturing firms are present than in those where imports are required. In addition, the versatility of microorganisms in substrate usage has led to an increase in the use of low-cost agricultural waste, including husks, straw, and fruit pulp, as substrates that could readily curtail production costs [72]. Although viability studies considering the economic practicality of microbial carotenoid production include factors that affect running costs (such as electricity and water tariffs incurred from production), research tends to focus on optimizable factors that affect microbial growth and product yield, such as media composition and temperature.

### 4.1. Growth Factor Effects on C_30_ Carotenoid Production

The main regulatory factors for carotenoid production in nonphotosynthetic bacteria are temperature, aeration, agitation, and culture media composition. Conversely, carotenoid synthesis by phototrophic bacteria is modulated by oxygen and light, with the bacteria producing less oxidized forms of carotenoids in oxygen-deprived environments and synthesizing ketocarotenoids in oxygenated environments [73,74,75]. Modifications in the quantity and quality of these factors have various effects on different species of microorganisms when initiating carotenoid production. *Rhodotorula glutinis* DFR-PDY, a yeast strain, produces higher carotenoid yields when using fructose as a carbon source, but exhibits greater cell growth in galactose-supplemented media [76]. Although the lactic acid bacteria (LAB) *L. plantarum* subsp. *plantarum* KCCP11226^T^ showed maximum cell growth in maltose, it produced the greatest C_30_ carotenoid 4,4′-diaponeurosporene yield in lactose-supplemented media. Nitrogen sources also play a role in microbial growth and carotenoid yield, with beef extract being optimal for both cell growth and 4,4′-diaponeurosporene production [31,77,78].

In lieu of conventional supplementation, the use of waste products for fermentation in carotenoid production is cost-effective and sustainable. Fermentation can be carried out in a solid state (SSF), or via submerged fermentation (SmF). IN SSF, the substrate consumption rate is slower, and fermentation requires a longer time and less moisture (such as in wheat bran and fruit pulp). In SmF, substrate consumption occurs rapidly, and the resupply of substrates, which include soluble sugars, molasses, and fruit juice, must be constant [79].

### 4.2. Abiotic Stresses for the Optimization of C_30_ Carotenoid Production

Various methods have been used to enhance carotenoid yield, including the application of stress factors, progressive nutrient deprivation, and recombinant techniques for the production of high-yield strains (Table 2) [44].

The processes employed in biotechnological applications depend on the stability and synthesis requirements of the desired products. Carotenoid production and cell growth are affected by the nutrient composition of the culture medium and culture conditions. For industrial production, the costs associated with substrates and the running costs of large-scale fermenters must be minimized while optimizing final product yields. Optimization studies that use response surface methodology often analyze the main factors for scaled-up production and maximize desired parameters, such as carotenoid production, while mitigating undesirables, such as high cost. An example of this is the optimization of nitrogen and carbon sources, particularly when standard media components are substituted by natural compounds or agricultural waste. This optimization reduces costs while also increasing sustainability, yielding environmentally friendly production methods [72].

Microbial growth or carotenoid synthesis can be maximized within a specific range of conditions. Environmental conditions optimal for cell growth are not necessarily optimal for carotenogenesis. In certain bacterial species, high temperatures are ideal, whereas in others, lower temperatures provide better carotenoid synthesis. This is also true for pH, salinity, and carbon and nitrogen sources. However, with biotechnological advancements, the effects of these modulating parameters on carotenoid production can be adjusted. Metabolically engineered strains of *E. coli* have been compared to determine the effect of recombinant hosts and carotenoid structure on carotenoid yield and ascertain whether heterologous carotenoid formation was strain-dependent.

The initial pH of the culture media influences bacterial cell growth and carotenoid production, but as the bacteria grow, the acidity/alkalinity levels of the media change due to by-products of bacterial metabolism [83], as is the case with LAB. In yeast cells, the initial decrease in pH is not permanent and the pH rises as the yeast proliferates and intensively produces carotenes, followed by stabilization of the pH values [84]. Acid or alkaline tolerance is highly strain-dependent, with certain microorganisms accumulating more carotenoids at neutral pH, while others exhibit optimum productivity in slightly acidic or alkaline conditions [85,86].

Temperature is an important factor in bacterial growth and carotenoid synthesis and affects biosynthetic pathways, stability, reactivity of substrates, enzymes, and the products involved in these pathways [87]. Temperature also plays a role in enzyme concentrations within biosynthetic pathways and in internal temperature regulation [88,89].

Aeration has a known effect on aerobic microorganisms, with agitation increasing the movement and availability of oxygen and nutrients within the culture. For aerobic microorganisms, culture aeration is important for the efficient supply of oxygen to the growing biomass, which in turn promotes carotenoid synthesis. Intensive aeration and agitation stimulate carotenoid production as long as the threshold at which the mechanical shear forces could cause cell damage is not crossed [90,91].

The substrate source and composition in the culture media also influence carotenoid production and yield. The most common carbon sources for carotenoid biosynthesis are sucrose and glucose. Cell growth maintenance requires available carbon, and there is a relationship between carotenoid production and carbon:nitrogen ratios in certain bacteria [92].

White-light irradiation has a positive effect on carotenoid synthesis in algae, fungi, and bacteria [93]. The carotenoid yield depends on the microorganism strain and light intensity. White light acts as a stimulatory inducer for cell growth, which then follows carotenoid accumulation, owing to increased enzyme activity [94]. White light increases carotenoid production by regulating biosynthetic genes in the pathway. High light intensity can restrict cell development and growth while inducing carotenoid synthesis [85].

Stress can be utilized to improve either cell growth or carotenoid production, and previous studies have shown that the two are not necessarily related or proportional. Different species respond differently to stress factors, either increasing or decreasing carotenoid concentrations [72]. Carotenoid accumulation can be enhanced at lower incubation temperatures [95], although carotenogenesis is not necessarily related to cell growth or biomass yield [72,96]. In microalgae, stress can stimulate carotenoid yields but production is limited by slow growth rates, required culture volumes, and bacterial contamination risks [97].

Salinity, oxidative stress, and pH have been used to increase carotenoid concentrations [10]. This increase is a result of microbial defense mechanisms triggered via gene regulation to modulate and alleviate the effects of stress factors [98]. However, while stress can trigger and stimulate carotenoid accumulation, stressful environments past the tolerance point will more likely trigger cell death than carotenoid accumulation.

## 5. Biosynthetic Pathway Engineering for Novel C_30_ Carotenoid Generation

**Figure 5:**
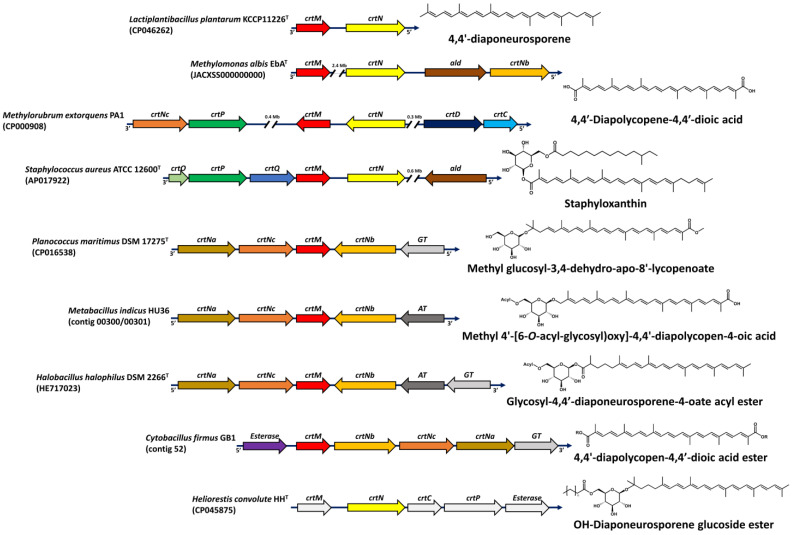
Gene clusters of 4,4′-diapocarotenoids and apo-8′-carotenoids from various microbial sources. White arrows represent putative genes in the biosynthetic pathway, including new and novel carotenoids.

Both natural and engineered extended pathways have been identified and developed for the synthesis of C_30_ carotenoids [99]. Metabolic engineering and directed evolution allow the development of new pathways for carotenoid production in noncarotenogenic microorganisms through the amalgamation of carotenoid genes from various sources. The enzymes expressed from these genes or gene clusters work in tandem to produce the desired compound. Additionally, the promiscuity of these carotenoid enzymes is based on end-group recognition rather than on the whole carotenoid structure (Figure 5) [100].

The genes involved in the synthesis of the C_30_ carotenoid skeleton are dehydrosqualene synthase (*crtM*) and dehydrosqualene desaturase (*crtN*) [14]. However, several novel C_30_ carotenoids have been created through combinatorial biosynthesis using enzymes from different microorganisms. For instance, C_40_ carotenoids were modified to produce cyclic C_30_ diapocarotenoids using lycopene cyclase, CrtY, which catalyzes the reaction on the ψ-end groups [101].

Various carotenoid structures can be obtained by manipulating biosynthetic pathways. Kim et al. [36] synthesized the acyclic, monocyclic, and bicyclic forms of C_30_ carotenoids from *E. coli* that had undergone directed evolution and a combination of biosynthetic processes. The extension of the diapolycopene pathway led to the formation of diaponeurosporene and the novel cyclic carotenoid, diapotorulene [58,99]. Following comparisons with other lycopene cyclase producers, Kim et al. [36] selected C_40_ CrtY from *Brevibacterium linens* (CrtYBL) to catalyze the cyclization of a saturated end of 4,4′-diaponeurosporene and produce the structurally novel monocyclic 4,4′-diapotorulene. CrtN evolution was used to optimize the 4,4′-diaponeurosporene or 4,4′-diapo-ζ-carotene biosynthesis pathways for cyclization to produce cyclic C_30_ carotenoids not found in nature. Modifications to the enzymes and pathways also led to the formation of other structurally novel compounds. These compounds include bicyclic C_30_ 4,4′-diapo-β-carotene; monocyclic C_30_ carotenoids harboring modified β-ionone rings, 7-hydroxy-4,4′-diapotorulene and 8-keto-4,4′-diapotorulene; monocyclic C_30_ carotenoids with modified acyclic ends, 4,4′-diapotorulen-4′-al and 4,4′-diapotorulen-4′-oic acid; and bicyclic compounds, 4,4′-diapo-β-cryptoxanthin, 4,4′-diapozeaxanthin,4,4′-diapoechinenone, and 4,4′-diapo-β-cryptoxanthin glucoside [36].

Tao et al. [37] identified a novel gene (*crtNb*) from *Methylomonas*, and its homologue in Staphylococcus, which converts 4,4′-diapolycopene to 4,4′-diapolycopene aldehyde, Additionally, they identified an aldehyde dehydrogenase gene (*ald*) involved in the oxidation of 4,4′-diapolycopene aldehyde to 4,4′-diapolycopene acid.

New acyclic carotenoids, diapolycopenedioc acid xylosylesters A–C, and the C_30_ aglycone, methyl 5-glucosyl-5,6-dihydro-apo-4,4′-lycopenoate, were produced by *Rubritalea squalenifaciens* and *P. maritimus* strains. The diapolycopenedioc acids xylosylesters A, B, and C had 2-acyl-D-xylose in their structures and were derivatives of the aglycone diapolycopenedioc acid [61]. In another study on pathway manipulation, diaponeurosporene was formed when CrtN introduced four double bonds into dehydrosqualene to produce a completely conjugated diapolycopene in recombinant *E. coli*, substituting the three-step desaturation [17]. Lee et al. [58] extended the acyclic C_30_ diaponeurosporene pathway using the C_40_ carotenoid enzymes spheroidene monooxygenase and lycopene cyclase to produce new oxygenated acyclic products and the novel cyclic C_30_ compound, diapotorulene.

Host strains also play a role in the biosynthetic pathway and final carotenoid structure, although the reason for this has not yet been determined. The acyclic C_30_ carotenoids diaponeurosporene and diapolycopene were produced by seven *E. coli* host strains (Top10, MG1655, MDS42, JM109, SURE, DH5a, and XL1-Blue). However, monocyclic diapotorulene was heavily strain-dependent and favored the SURE *E. coli* strain in a manner similar to that of heterologous lycopene [57].

With regard to bioengineered pathways, engineered microbial hosts designed for high carotenoid yields require optimization of their isoprenoid precursor pool. Moreover, microbes must be able to contain lipophilic carotenoids and the expression of carotenogenic genes need to be modulated for the efficient transformation of precursors into target compounds. Optimization of the precursor pool requires increasing the isoprenoid flux via overexpression of enzymes in the nonmevalonate isoprenoid pathway. Balancing isoprenoid expression and carotenoid-synthesizing enzymes enhances carotenoid production by alleviating the effects of growth inhibition caused by enzyme overexpression.

## 6. Future Predictions and Expectations

The limitations of microbial carotenoid production are mainly insufficient yields and poor consumer acceptance, especially with a bias against genetically modified organisms. Industry regulations in the food, feed, and nutraceutical fields may also hinder their commercial availability. Additionally, production inconsistency and optimization requirements may hamper their ability to compete with plant extractions and chemical synthetic sources [44].

Biotechnological advances will likely lead to the development of carotenoids with enhanced antioxidant capacities, capable of tackling metabolic syndrome-associated conditions and debilitating diseases. As research progresses toward the industrialized and commercial microbial production of carotenoids, it is imperative that consumer acceptance and industry regulations be considered.

With the discovery of carotenoid biosynthetic pathways in different microorganisms and the advancement of metabolic engineering, a vast number of C_30_ carotenoids will likely be added to the current pool of known carotenoid compounds. Gene editing and biosynthetic pathway expansion have led to novel C_30_ carotenoids and intermediates with different characteristics from their counterparts and derivatives. The formation of carotenoids with superior antioxidant activity could propel further developments in microbial carotenoid production and consumer acceptance.

## 7. Conclusions

The review highlights the antioxidant potential of C_30_ carotenoids from various microbial sources and the synthesis of novel C_30_ carotenoid compounds through metabolic engineering. It validates the prospect of commercialization of these compounds as alternatives for the more common C_40_ carotenoids that take up a majority of the global market. The synthesis of C_30_ carotenoids that possess similar or greater antioxidant activities than C_40_ carotenoids would be a worthwhile path for novel marketable antioxidant products in the sectors of food, feed, cosmetics, and pharmaceuticals. Microbial synthesis provides faster and safer production conditions with nontoxic waste by-products as opposed to chemical synthesis. Additionally, the simplicity of genetic manipulation and process optimization can aid in cutting costs that are detrimental to large-scale production. The isolation of 4,4′-diaponeurosporene from *Lactiplantibacillus plantarum* subsp. *plantarum* is particularly noteworthy because of the “generally regarded as safe” (GRAS) status of the microorganism and its role as a probiotic. The supplementation of foods with probiotics that can synthesize carotenoids with high antioxidant potential can offer great health benefits by promoting digestive health in addition to preventing cellular damage through the reduction of oxidative stress in the human body. More research is required to improve and optimize the development and applications of microbial carotenoid sources, by obtaining more thorough knowledge on even the less common carotenoid types.

## Figures and Tables

**Table 2:** Effects of applied factors on C_30_ carotenoid production.

Controlled Factor	Action	Effect	Ref.
Oxidative stress	Diphenylamine and duroquinone application	Complete inhibition of C_30_ carotenoids	[80]
Oxidative stress	Diphenylamine application	Complete inhibition of C_30_ carotenoids	[16]
Oxidative stress	Hydrogen peroxide exposure	5.9-fold increase in carotenoid production	[29]
Temperature	Low temperature	Increased 4,4′-diaponeurosporene antioxidant activity– DPPH: 1.7-fold vs BHT; ABTS: 7.5-fold vs BHT; FRAP: 8-fold vs BHT	[31]
Recombinant technique	Constitutive lac promoter	Increased 4,4′-diapolycopene production	[81]
Recombinant technique	Optimizing engineered pathways in heterologous host	Increased 4,4′-diapolycopene and 4,4′-diaponeurosporene production	[82]
Recombinant technique	Gene coexpression	20-fold increase in 4,4′-diapolycopene dialdehyde production	[37]
Recombinant technique	Farnesyl diphosphate (FDP) synthase overexpression	3- to 5-fold increase in diapotorulene-to-diaponeurosporene ratio	[58]

## Data Availability

Not applicable.
